# Predicting EGFR mutation, ALK rearrangement, and uncommon EGFR mutation in NSCLC patients by driverless artificial intelligence: a cohort study

**DOI:** 10.1186/s12931-022-02053-2

**Published:** 2022-05-27

**Authors:** Xueyun Tan, Yuan Li, Sufei Wang, Hui Xia, Rui Meng, Juanjuan Xu, Yanran Duan, Yan Li, Guanghai Yang, Yanling Ma, Yang Jin

**Affiliations:** 1 Department of Respiratory and Critical Care Medicine, NHC Key Laboratory of Pulmonary Diseases, Union Hospital, Tongji Medical College, Huazhong University of Science and Technology, 1277 Jiefang Avenue, Wuhan, 430022 China; 2 Department of Oncology, Renmin Hospital of Wuhan University, Wuhan University, Wuhan, China; 3 Union Oncology Hospital, Tongji Medical College, Huazhong University of Science and Technology, Wuhan, China; 4 School of Public Health, Tongji Medical College, Huazhong University of Science and Technology, Wuhan, China; 5 Department of Pathology, Union Hospital, Tongji Medical College, Huazhong University of Science and Technology, Wuhan, China; 6 Department of Thoracic Surgery, Union Hospital, Tongji Medical College, Huazhong University of Science and Technology, Wuhan, China

**Keywords:** Non-small cell lung cancer, Serum tumor markers, Epidermal growth factor receptor, Anaplastic lymphoma kinase, Artificial intelligence, Deep learning, Machine learning

## Abstract

**Background:**

Timely identification of epidermal growth factor receptor (EGFR) mutation and anaplastic lymphoma kinase (ALK) rearrangement status in patients with non-small cell lung cancer (NSCLC) is essential for tyrosine kinase inhibitors (TKIs) administration. We aimed to use artificial intelligence (AI) models to predict EGFR mutations and ALK rearrangement status using common demographic features, pathology and serum tumor markers (STMs).

**Methods:**

In this single-center study, demographic features, pathology, EGFR mutation status, ALK rearrangement, and levels of STMs were collected from Wuhan Union Hospital. One retrospective set (N = 1089) was used to train diagnostic performance using one deep learning model and five machine learning models, as well as the stacked ensemble model for predicting EGFR mutations, uncommon EGFR mutations, and ALK rearrangement status. A consecutive testing cohort (n = 1464) was used to validate the predictive models.

**Results:**

The final AI model using the stacked ensemble yielded optimal diagnostic performance with areas under the curve (AUC) of 0.897 and 0.883 for predicting EGFR mutation status and 0.995 and 0.921 for predicting ALK rearrangement in the training and testing cohorts, respectively. Furthermore, an overall accuracy of 0.93 and 0.83 in the training and testing cohorts, respectively, were achieved in distinguishing common and uncommon EGFR mutations, which were key evidence in guiding TKI selection.

**Conclusions:**

In this study, driverless AI based on robust variables could help clinicians identify EGFR mutations and ALK rearrangement status and provide vital guidance in TKI selection for targeted therapy in NSCLC patients.

**Supplementary Information:**

The online version contains supplementary material available at 10.1186/s12931-022-02053-2.

## Background

Precise classification of lung cancer types is vital for selecting the proper treatment with the best efficacy. With the rapid evolution of molecular targeted therapy, the survival of non-small cell lung cancer (NSCLC) patients with mutations, such as epidermal growth factor receptor (EGFR) and anaplastic lymphoma kinase (ALK) mutations, has improved significantly. Previous studies have reported that 10–20% of Caucasian and at least 50% of Asian non-squamous NSCLC patients harbored activating EGFR mutations [1–4], and the high absolute number of ALK-positive NSCLC patients, mainly with the adenocarcinoma subtype, was due to 3–7% of NSCLCs harboring ALK rearrangements [5]. Given that a large number of lung cancer patients are subject to mutations, accurately identifying these patients is essential so that tyrosine kinase inhibitors (TKIs) can be administered in a timely manner to improve their outcomes. Hitherto, as standard first-line treatments, TKIs have been developed for multiple generations [6]. TKI treatment has been proven to significantly improve response rates and prolong progression-free survival (PFS) in EGFR-mutated and ALK-positive NSCLC patients [7, 8]. To date, the gold standard for measuring EGFR and ALK status is mutational sequencing of tumor tissue acquired from biopsy. In the meantime, next-generation sequencing (NGS) technologies provide great help in understanding the genomic profiles of NSCLC [9]. However, the invasion, low efficiency, and relatively high cost of tumor biopsy constrain its frequent use in patients, as mutational status may change during therapy and progression [10]. In addition, tumor tissue is not available for approximately 49% of advanced or metastatic NSCLC patients [11]. Therefore, the development of a noninvasive and more convenient method to predict EGFR and ALK mutation status is of great interest.

Some studies have investigated the relationship between clinical image features, including histopathology images, computed tomography (CT) images, PET/CT images, and EGFR mutation status in NSCLC [12–14]. An EGFR mutation prediction model constructed based on ^18^F-FDG PET/CT radiomic features had an area under the curve (AUC) of 0.87 [15]. The feasibility of liquid biopsies has also been highlighted recently; ctDNA, circulating tumor cells (CTCs), and exosomes derived from tumor cells existing in body fluids have been found to be closely related to somatic alterations of tumors [16, 17]. Krug et al. indicated that combining exosomal RNA and circulating tumor DNA in the plasma of patients with NSCLC increased the sensitivity of EGFR mutation detection [11]. Our previous study suggested that serum tumor markers (STMs) integrated with other clinical factors could be a valuable noninvasive tool for predicting EGFR mutations and ALK positivity in NSCLC patients [18]. Several clinical tumor markers, such as carcinoembryonic antigen (CEA), cytokeratin 19 fragments (CYFRA 21-1), carbohydrate antigen 125 (CA-125), and carbohydrate antigen 19-9 (CA-199), have been shown to be valuable for the diagnosis of lung cancer and as predictors of survival in NSCLC patients [19, 20]. Furthermore, the value of STMs in predicting immunotherapy efficacy in NSCLC patients is being studied and has shown great potential [21, 22]. Hence, STMs are attractive tools for cancer studies because they are easily obtained as clinical indicators. STMs are of great significance in diagnosing lung lesions, and the fee for this analysis is provided by the health insurance in China.

To assist clinicians, artificial intelligence (AI) has been widely applied in the medical field, and its encouraging performance presents great hope in the current era of precision medicine. The application of machine learning (ML), an important subfield of AI, is growing rapidly in medicine [23, 24]. ML methods have been used to solve various problems in genomics and genetics, such as distinguishing between different disease phenotypes [25]. A previous study used a support vector machine (SVM) algorithm to establish a multiclass classifier to diagnose multiple common adult malignancies. The overall classification accuracy of the classifier was 78%, far exceeding the accuracy of random classification (9%) [26]. Mu et al. reported an ^18^F-FDG-PET/CT-based EGFR-deep learning score that can provide decision support for NSCLC treatment with TKIs or immune checkpoint inhibitors (ICIs) [27].

Based on our previous work, we aimed to use AI combined with STMs and other clinical factors to predict EGFR mutations, common and uncommon EGFR mutations, and ALK rearrangement status in NSCLC patients. Five ML models, namely distributed random forest (DRF), gradient boosting machine (GBM), generalized linear models (GLM), extreme gradient boosting (XGBoost), extremely randomized trees (XRT), and deep learning (DL) model, as well as the stacked ensemble model, were developed and evaluated simultaneously. All models were validated in the testing cohort, and the most appropriate model was used by comparing their performance measures.

## Methods

### Study population and data collection

This single-center cohort study consisted of a two-step approach (training and testing assessment) that included NSCLC patients in a published cohort (EJC cohort) [18] and model validation in a subsequent recruited cohort. The training cohort enrolled 1089 NSCLC patients displayed in a previously published cohort from January 2012 to December 2016 at Wuhan Union Hospital, Huazhong University of Science and Technology. To investigate the external validity, a consecutive set of 1464 NSCLC patients at Wuhan Union Hospital from January 2017 to December 2019 was used as the testing cohort. Patients at the first onset of NSCLC were recruited based on accurate diagnostic criteria according to international guidelines. Consistent data collected for each study participant with proven NSCLC included demographic features (age, sex, and smoking history) and results of STMs, as well as other examinations. Nonsmokers were defined as never-smokers or those who smoked less than 100 cigarettes in their lifetime. The remaining patients were defined as ever-smokers. Before any anticancer therapy, blood samples of all enrolled patients were obtained through peripheral venipuncture, and a commercial chemiluminescence immunoassay kit (Abbott Laboratories, I4000, America) was used to detect STMs.

The inclusion criteria were as follows:

(1) At least one of twelve STMs, including CEA, squamous cell carcinoma antigen (SCC), prostate specific antigen (PSA), free prostate specific antigen (FPSA), CYFRA 21-1, neuronspecific enolase (NSE), alpha fetoprotein (AFP), CA 125, CA 19-9, CA 15-3, ferritin (FERR), and CA 72-4 must have been tested; (2) EGFR mutation and ALK rearrangement status must have been tested sequentially within two weeks.

The exclusion criteria were as follows:

(1) Patients received treatment before EGFR mutation and ALK rearrangement status detection; (2) the results of pathological examination from different tumor sites suggested different pathological types or could not be categorically classified as a single pathological type; and (3) patients had a history of another cancer.

This study was conducted in accordance with the International Council for Harmonization Guidelines for Good Clinical Practice and the Declaration of Helsinki. And was registered on the Clinical Trials website (No. NCT04005677).

### Identification of EGFR mutation

The method used to detect EGFR mutations was the same as that in a previous study [18], roughly outlined as follows: collecting histological specimens of primary tumors, metastatic lymph nodes or organs, and cytological specimens of pleural or pericardial effusion; fixing specimens in 10% neutral buffered formalin and embedding them in paraffin; and then performing experiments using the QIAamp DNA formalin-fixed paraffin-embedded (FFPE) Tissue Kit (Qiagen NV, Venlo, Netherlands), Mx3000PTM real-time PCR system (Stratagene, La Jolla, USA), and EGFR 29 Mutations Detection Kit (Amoy Diagnostics, Xiamen, People’s Republic of China) to detect any exon mutations. The tumor was identified as an “EGFR mutant” if an exon mutation was detected. Somatic mutations in the tyrosine kinase domain of EGFR, which is an oncogenic mechanism, can dysregulate the tyrosine kinase (TK) activity of EGFR [28]. An exon 19 deletion (Del19) and an L858R point mutation are major EGFR mutations that are sensitive to EGFR TKIs, and 80–90% of patients with EGFR-mutated NSCLC have one of these two types of EGFR mutations [29]. These two types of EGFR mutations are common. Uncommon EGFR mutations in NSCLC patients involving exon 20, including T790M and exon 20 insertions, are not sensitive to the first-generation EGFR TKIs. The EGFR T790M mutation manifests as a single amino acid substitution from threonine to methionine at position 790 in the wild-type EGFR kinase domain, which is the most prevalent resistance mutation in first- and second-generation EGFR-TKI [30, 31]. Insertions in exon 20 represent a combination of in-frame insertions and/or duplications of 1–7 amino acids between the α-C helix and the 762–774 amino acid sequence [32, 33]. T790M, exon 20 insertions, and other uncommon EGFR mutations such as G719X, L861Q, and S768I are categorized as uncommon EGFR mutations.

### Identification of ALK gene rearrangement

Ventana immunohistochemistry (IHC) was performed using tumor specimens to confirm ALK rearrangement by analyzing formalin-fixed paraffin-embedded (FFPE) tissues, as described in our previous study [18]. ALK IHC positivity is characterized by strong granular cytoplasmic staining in any percentage of tumor cells; otherwise, the sample was deemed ALK-negative. ALK IHC has a high sensitivity and specificity for detecting ALK rearrangements [34, 35].

### Machine learning and deep learning classifiers

**Fig. 1:**
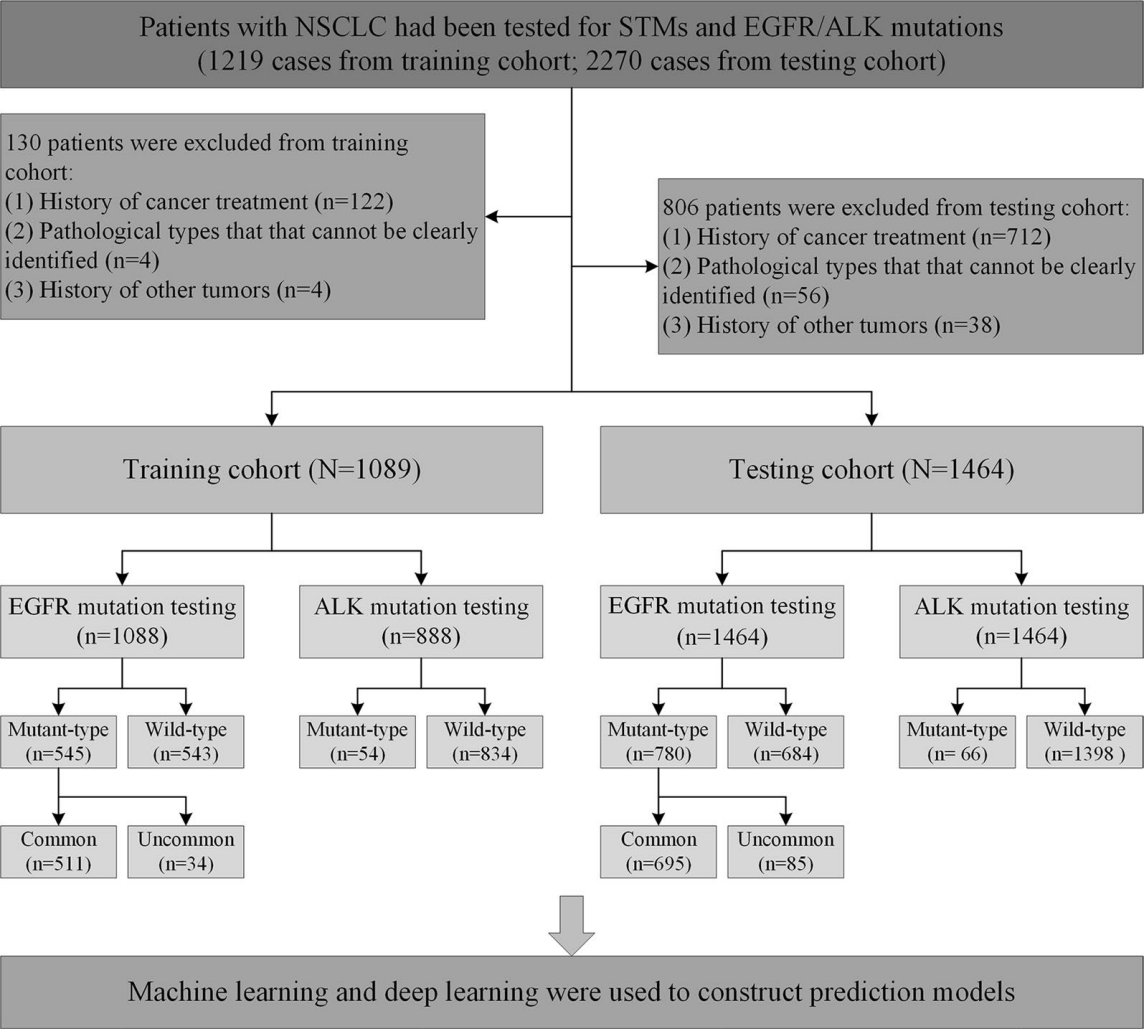
Study design and patient selection

In this study, we used model-developing methods based on a previous study by Li et al. [36]. DL and five ML models, including the GBM, XGBoost, XRT, DRF, and GLM, were used to create predictive models for identifying EGFR mutations, common and uncommon EGFR mutations, and ALK rearrangements in NSCLC patients. The details of the ML and DL algorithms are provided in the Additional file 1: Supplementary Methods. By combining the above models, stacked ensemble models with optimal performance were constructed. All the models were trained and tested for both cohorts. The workflow for training and testing candidate predictive models using computational algorithms is shown in Fig. 1.

The major strides in the development of user-friendly ML software that can be used by non-experts using H2O software is the Automatic Machine Learning (AutoML), which automates the process of training a large selection of candidate models. H2O’s AutoML can be used for automating the ML workflow, which includes the automatic training and tuning of many models within a user-specified time limit. For other supported methods, AutoML performs a hyperparameter search over various H2O algorithms to obtain the best model. The construction of stacked ensembles was based on all previously trained models and the best model for each family. In most cases, stacked ensemble models are the top performing models in the AutoML Leaderboard, which are automatically trained on collections of individual models to produce highly predictive ensemble models. Scikit-learn feature importance plots and variable importance heatmaps of all candidate models were generated through the matplotlib package built in AutoML, and all plots used for model interpretation were saved in the results directory.

### Statistical analysis

Continuous variables were compared between groups using either Student’s t-test or Mann–Whitney U test, as appropriate, and categorical data were compared using the chi-square test or Fisher’s exact test. The AUC of the receiver operator characteristic (ROC), specificity, sensitivity, positive predictive value (PPV), and negative predictive value (NPV) were calculated to evaluate the diagnostic performance of the models in predicting EGFR mutation status. We used R version 3.6.1 and SPSS 20.0 for the statistical analysis. For both sides, *P* < 0.05 with a 95% confidence interval was regarded as statistically significant.

## Results

### Patients’ clinical characteristics

A total of 2553 NSCLC patients were included in both cohorts. A total of 1089 NSCLC patients from the EJC cohort[18] as the training cohort and 1464 consecutive NSCLC patients from January 2017 to December 2019 at Wuhan Union Hospital as the testing cohort were enrolled in the analysis (Fig. 1). The clinical characteristics of the training cohort are presented in Table 1. EGFR mutations were identified in 780 patients (53.28%), with a median age of 60 years (range 31–86 years). Of the 1464 patients tested for ALK rearrangement, 66 (4.51%) were positive for ALK, with a median age of 54 years (range 27–81 years).

**Table 1:** Association between clinical characteristics and EGFR and ALK status in the testing cohort

Characteristics	EGFR			ALK		
	Wild-type	Mutant-type	P value	Wild-type	Mutant-type	P value
Age, years			0.390			< 0.001
Median	61	60		61	54	
Range	23–88	31–86		23–88	27–81	
Gender			< 0.001			0.337
Female	495 (72.37)	321 (41.15)		783 (56.01)	33 (50.00)	
Male	189 (27.63)	459 (58.85)		615 (43.99)	33 (50.00)	
Smoking history			< 0.001			0.121
Never-smoker	345 (50.51)	635 (81.41)		930 (66.57)	50 (75.76)	
Ever-smoker	338 (49.49)	145 (18.59)		467 (33.43)	16 (24.24)	
Pathology			< 0.001			0.036
Adenocarcinoma	585 (85.53)	753 (96.54)		1273 (91.06)	65 (98.48)	
Non-adenocarcinoma	99 (14.47)	27 (3.46)		125 (8.94)	1 (1.52)	
AFP			0.104			1.000
Negative	538 (98.18)	642 (99.23)		1126 (98.69)	54 (100.00)	
Positive	10 (1.82)	5 (0.77)		15 (1.31)	0 (0.00)	
CEA			0.014			0.060
Negative	341 (50.52)	342 (44.07)		645 (46.54)	38 (58.46)	
Positive	334 (49.48)	434 (55.93)		741 (53.46)	27 (41.54)	
CA125			< 0.001			0.164
Negative	319 (48.41)	458 (59.87)		748 (54.96)	29 (46.03)	
Positive	340 (51.59)	307 (40.13)		613 (45.04)	34 (53.97)	
CA19-9			0.031			0.530
Negative	493 (75.61)	610 (80.37)		1055 (78.32)	48 (75.00)	
Positive	159 (24.39)	149 (19.63)		292 (21.68)	16 (25.00)	
CA15-3			0.195			< 0.001
Negative	451 (75.04)	542 (78.10)		962 (77.64)	31 (55.36)	
Positive	150 (24.96)	152 (21.90)		277 (22.36)	25 (44.64)	
FERR			< 0.001			0.135
Negative	210 (55.56)	289 (68.16)		471 (61.65)	28 (73.68)	
Positive	168 (44.44)	135 (31.84)		293 (38.35)	10 (26.32)	
CA72-4			0.010			0.209
Negative	289 (72.43)	369 (79.87)		630 (76.83)	28 (68.29)	
Positive	110 (27.57)	93 (20.13)		190 (23.17)	13 (31.71)	
PSA			0.610			0.711
Negative	262 (91.93)	165 (93.22)		403 (92.22)	24 (96.00)	
Positive	23 (8.07)	12 (6.78)		34 (7.78)	1 (4.00)	
FPSA			0.436			1.000
Negative	272 (95.44)	166 (93.79)		414 (94.74)	24 (96.00)	
Positive	13 (4.56)	11 (6.21)		23 (5.26)	1 (4.00)	
SCC			< 0.001			0.178
Negative	472 (76.62)	636 (90.34)		1063 (84.23)	45 (77.59)	
Positive	144 (23.38)	68 (9.66)		199 (15.77)	13 (22.41)	
CYFRA 21-1			< 0.001			0.137
Negative	216 (35.64)	332 (47.91)		519 (41.75)	29 (51.79)	
Positive	390 (64.36)	361 (52.09)		724 (58.25)	27 (48.21)	
NSE			0.034			0.472
Negative	212 (44.17)	268 (50.85)		460 (47.92)	20 (42.55)	
Positive	268 (55.83)	259 (49.15)		500 (52.08)	27 (57.45)	
TTF-1			< 0.001			0.145
Negative	129 (20.48)	14 (2.04)		140 (11.13)	3 (5.08)	
Positive	501 (79.52)	673 (97.96)		1118 (88.87)	56 (94.92)	
Napsin A			< 0.001			0.401
Negative	121 (28.67)	19 (5.23)		135 (18.10)	5 (12.82)	
Positive	301 (71.33)	344 (94.77)		611 (81.90)	34 (87.18)	
CK-7			0.006			1.000
Negative	19 (5.79)	3 (1.27)		21 (3.94)	1 (3.13)	
Positive	309 (94.21)	234 (98.73)		512 (96.06)	31 (96.88)	
Ki67			0.083			0.528
Negative	37 (28.03)	28 (40.00)		60 (31.58)	5 (41.67)	
Positive	95 (71.97)	42 (60.00)		130 (68.42)	7 (58.33)	

Values presented are n (%) unless otherwise noted

*EGFR* epidermal growth factor receptor; *ALK* anaplastic lymphoma kinase; *AFP* alpha fetoprotein; *CEA* carcinoembryonic antigen; *CA* carbohydrate antigen; *FERR* ferritin; *PSA* prostate specific antigen; *FPSA* free prostate specific antigen; *SCC* squamous cell carcinoma antigen; *CYFRA 21-1* soluble fragment of cytokeratin 19; *NSE* neuron-specific enolase; *TTF-1* thyroid transcription factor-1; *CK-7* cytokeratin-7

The training cohort was used to explore the predictors of EGFR and ALK mutations in our previous study [18]. Adenocarcinoma (ADC), never-smoker status, and negative CA 125 and SCC results were predictors of EGFR mutations, while younger age and never-smoker status were independent predictors of ALK rearrangement. The predictors showed similar discriminative power in differentiating EGFR mutations or ALK rearrangement in the testing cohort, as reflected by an AUC of 0.669 (*P* < 0.001) or 0.654 (*P* < 0.001) (Additional file 2: Fig. S1A–B). These results demonstrated that our data were largely consistent and reliable for further analysis.

### Development and validation of deep learning and machine learning models to predict EGFR mutation or ALK rearrangement status

**Fig. 2:**
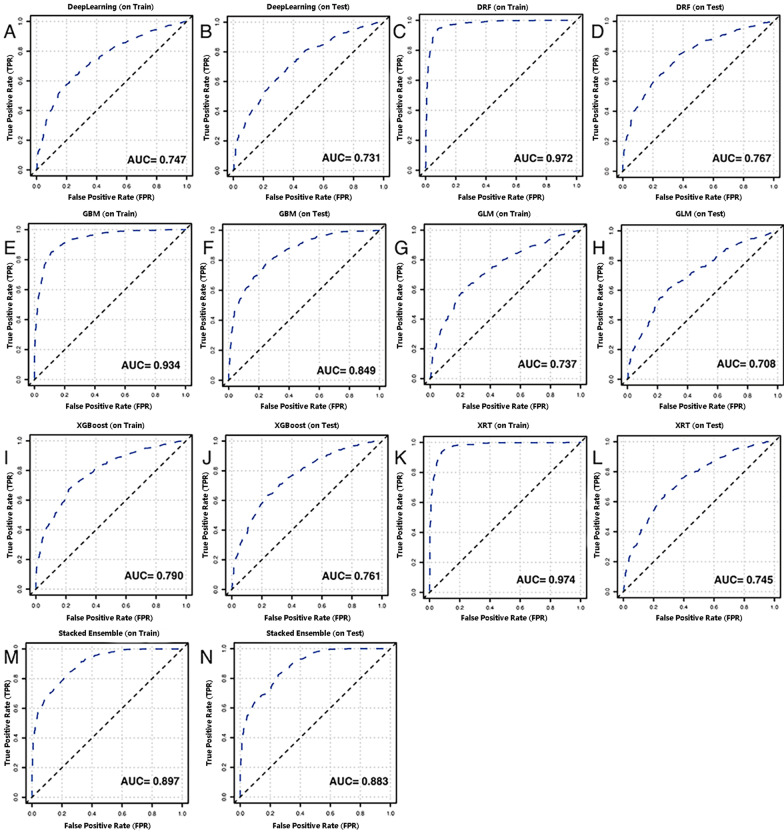
Discrimination of the computational algorithms for discrimination of EGFR mutant status in the training cohort and the testing cohort. **A**–**B** Deep leaning model; **C**–**D** DRF model; **E**–**F** GBM model; **G**–**H** GLM model; **I**–**J** XGBoost model; **K**–**L** XRF model; **M**–**N** Stacked Ensemble model

**Fig. 3:**
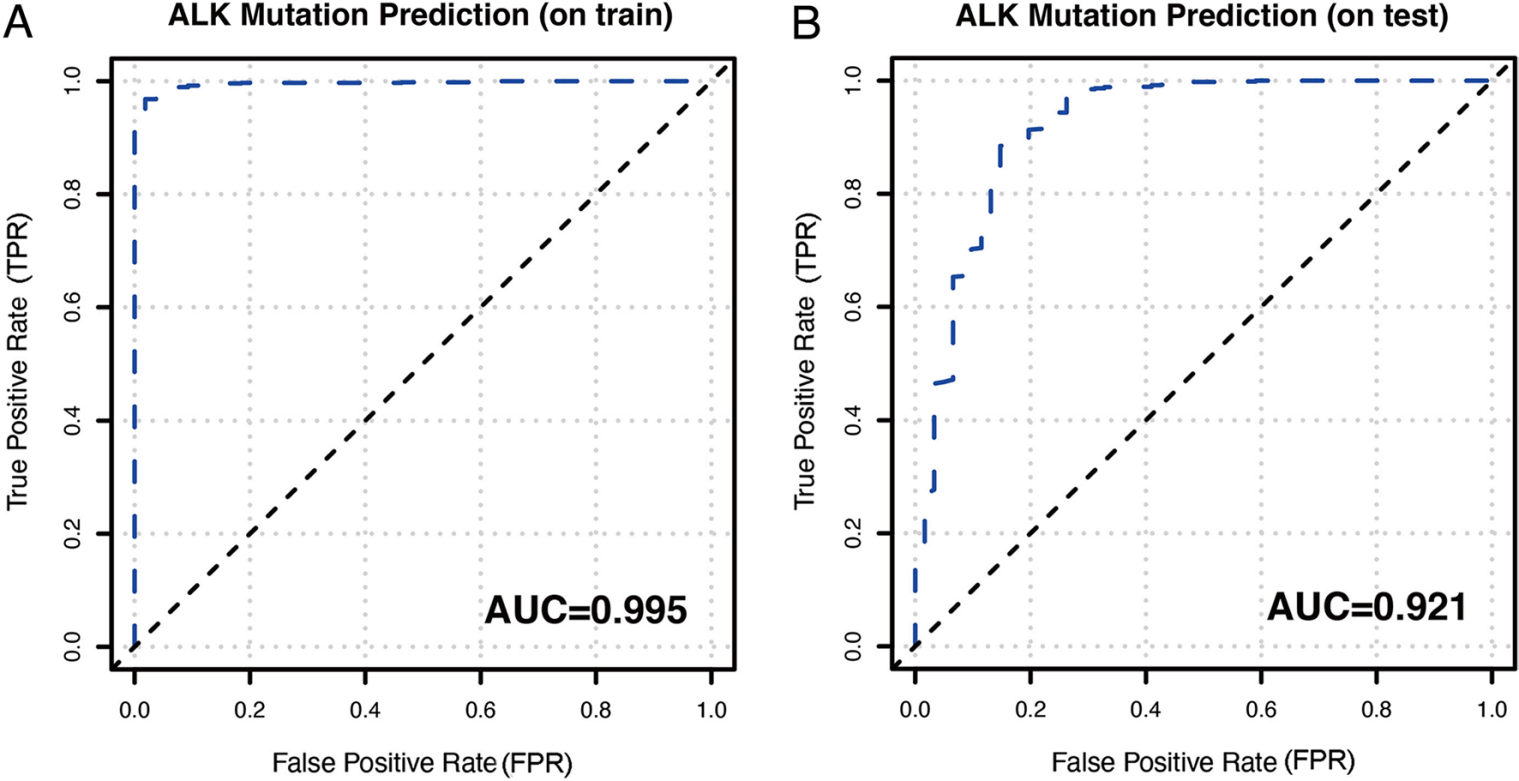
Discrimination of Stacked Ensemble model for discrimination of ALK rearrangement in the training cohort (**A**) and the testing cohort (**B**)

Although the above factors played an important role in the identification of EGFR mutation status or ALK rearrangement, the methods had a relatively low AUC value. To make the best use of these clinical data, one DL and five ML models were utilized to distinguish EGFR status in the training cohort. As shown in Fig. 2, the AUCs of the above six models were 0.747 for the DL model, 0.972 for DRF, 0.934 for GBM, 0.737 for GLM, 0.790 for XGBoost, and 0.974 for XRF. The DRF and XRF models exhibited preferable discernibility. In the testing cohort, the AUCs of the six models were 0.731 for the DL model, 0.767 for DRF, 0.849 for GBM, 0.708 for GLM, 0.761 for XGBoost, and 0.745 for XRF. The GBM model achieved the best predictive ability for differentiating EGFR mutation status. Finally, a stacked ensemble model combining the above six models was used to create the best prediction algorithms. The stacked ensemble model showed favorable discriminative power. The AUC of the stacked ensemble model were 0.897 and 0.883 for the training and testing cohorts, respectively (Fig. 2). The sensitivity, specificity, accuracy, PPV, and NPV of the stacked ensemble model were 0.835, 0.677, 0.578, 0.886, and 0.732, respectively, in the training cohort and 0.856, 0.680, 0.638, 0.877, and 0.750, respectively, in the testing cohort (Table 2). ALK rearrangements were distinguished using another stacked ensemble model following the same pipeline. As shown in Fig. 3, the AUC of the stacked ensemble model were 0.995 and 0.921 for the training and testing cohorts (Fig. 3A, B).

**Table 2:** Performance measures of the stacked ensemble model for prediction two classifications of EGFR mutation

Cohort	Sensitivity	Specificity	Accuracy	PPV	NPV
The training cohort	0.835	0.677	0.578	0.886	0.732
The testing cohort	0.856	0.680	0.638	0.877	0.750

*EGFR* epidermal growth factor receptor; *PPV* positive predictive value; *NPV* negative predictive value

In order to illustrate the impact of pathological subtype on the prediction for EGFR mutation by the AI model, the same stacked ensemble model was re-tested in adenocarcinoma-only group in the training and testing cohort, respectively. This model showed similar discriminative performance in adenocarcinoma-only group, as reflected by an AUC of 0.873 among adenocarcinoma cases in the training cohort and an AUC of 0.820 among adenocarcinoma cases in the testing cohort (Additional file 3: Fig. S2A, B).

### Most informative parameters of deep learning and five machine learning models to predict EGFR mutation or ALK rearrangement status

To investigate the internal mechanism of different clinical features on the discriminative abilities of EGFR status, the five most informative parameters selected from high to low by DL and five ML models are displayed in Additional file 4: Fig. S3A–F. Patients with ADC are more prone to EGFR mutations. Pathology ranked first in the DL and GLM models. However, age ranked first in the DRF and XRT models, and smoking history ranked first in the GBM and XGBoost models, respectively (Additional file 1: Fig. S3A–F).

**Fig. 4:**
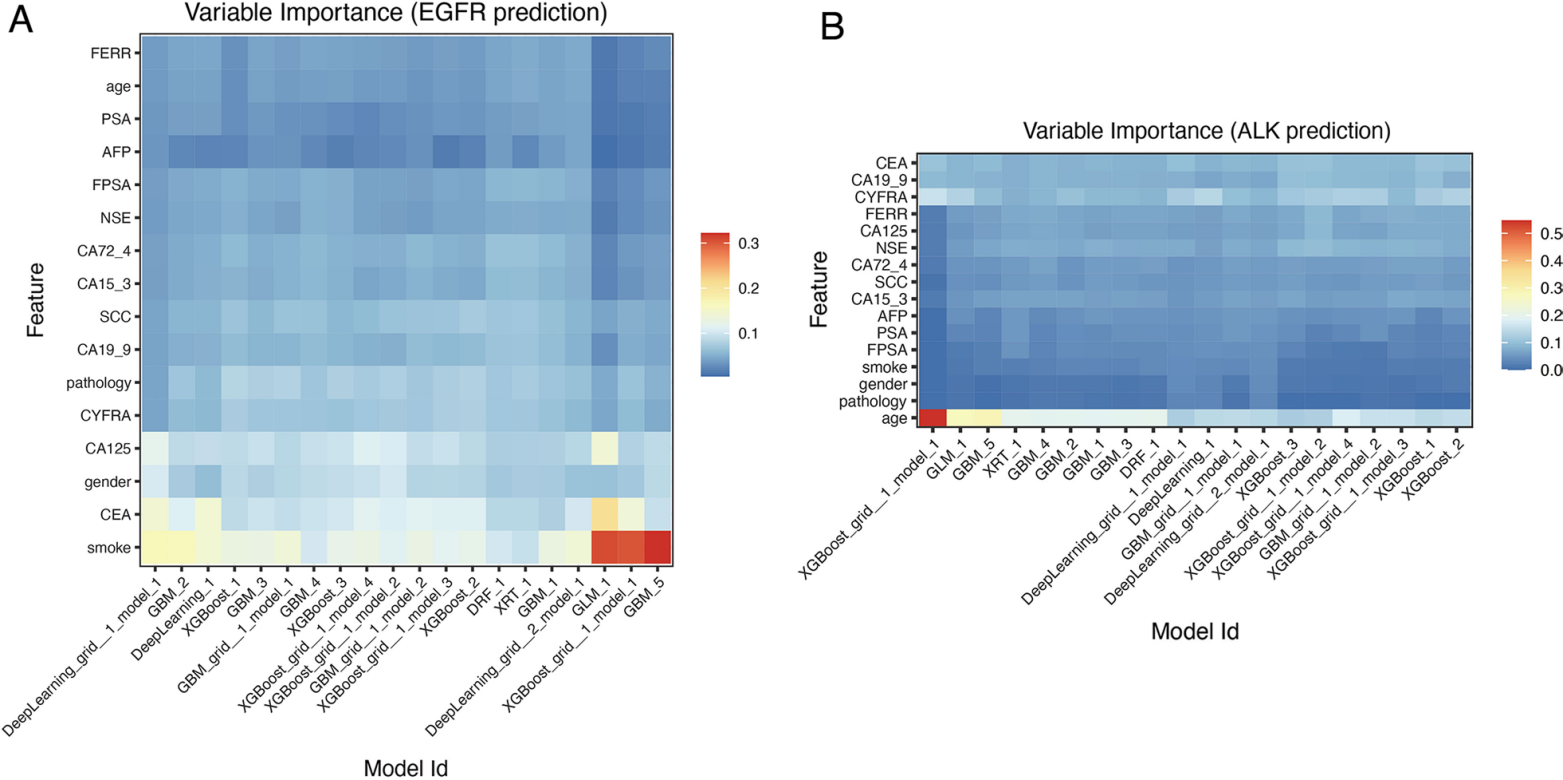
The importance of differential clinical parameters in different computational algorithm models. The AutoML randomly generated twenty algorithms based on deep learning model and five machine learning model, and the twenty models were interpreted using feature importance plots through the matplotlib package built in the software. **A** Variable importance for EGFR prediction. **B** Variable importance for ALK prediction

The importance of each clinical feature calculated by AutoML in different models and the correlations between different models to predict EGFR mutation or ALK rearrangement status are shown in Fig. 4 through a heatmap. As shown in Fig. 4A, smoking history, CEA levels, and gender provided important information for predicting EGFR mutations. As shown in Fig. 4B, age, pathology, and gender provided important information for predicting ALK rearrangement.

### Deep learning and machine learning models to predict uncommon EGFR mutations

**Fig. 5:**
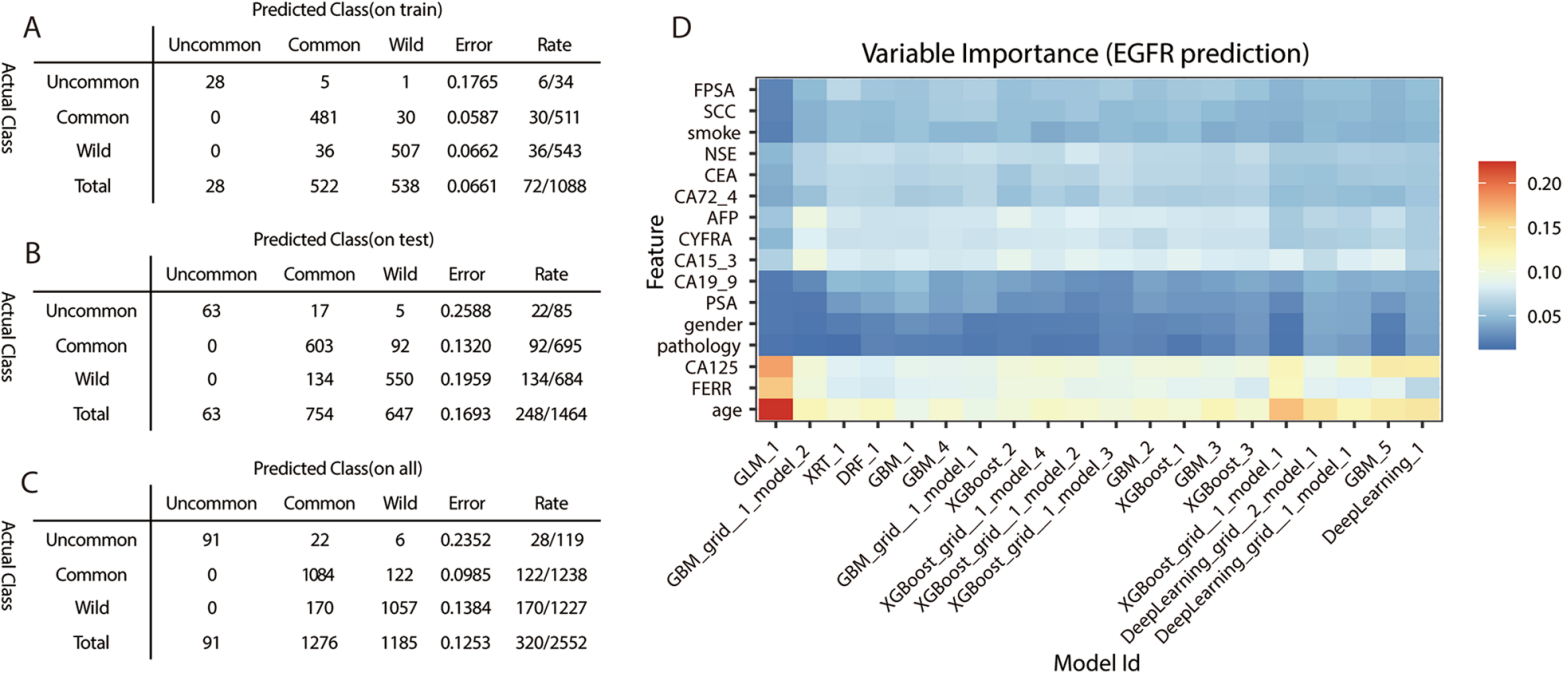
Stacked Ensemble model to distinguish common and uncommon EGFR mutations. **A**–**C** Discrimination of Stacked Ensemble model for identification of common and uncommon EGFR mutations in the training cohort, the testing cohort and total patients, respectively. **D** The importance of differential clinical parameters in the Stacked Ensemble model. The AutoML randomly generated twenty algorithms based on deep learning model and five machine learning model, and the twenty models were interpreted using feature importance plots through the matplotlib package built in the software

Common EGFR mutations, including Del19 and L858R, were sensitive to all three generations of EGFR-TKIs, whereas uncommon EGFR mutations including T790M, exon 20 insertions, and others showed varying degrees of sensitivity to EGFR-TKIs. Patients with some uncommon EGFR mutations may response poorly to first-generation TKIs. Therefore, specific EGFR mutation types may provide essential information for clinical decision making. Here, AI models were used to identify common (Del19 and/or L858R), uncommon EGFR mutations, and wild status in the training cohort, testing cohort, and all patients. The overall accuracy in the training cohort, testing cohort, and total patients was 0.93, 0.83, and 0.87, respectively (Fig. 5A–C). The importance of each clinical feature calculated by AutoML in different models and the correlations between different models to predict the three classifications of EGFR mutations are shown in Fig. 5D. Age, FERR levels, and CA125 levels provided important information for the six models.

### Deep learning and machine learning models to predict EGFR mutation and ALK rearrangement status

**Fig. 6:**
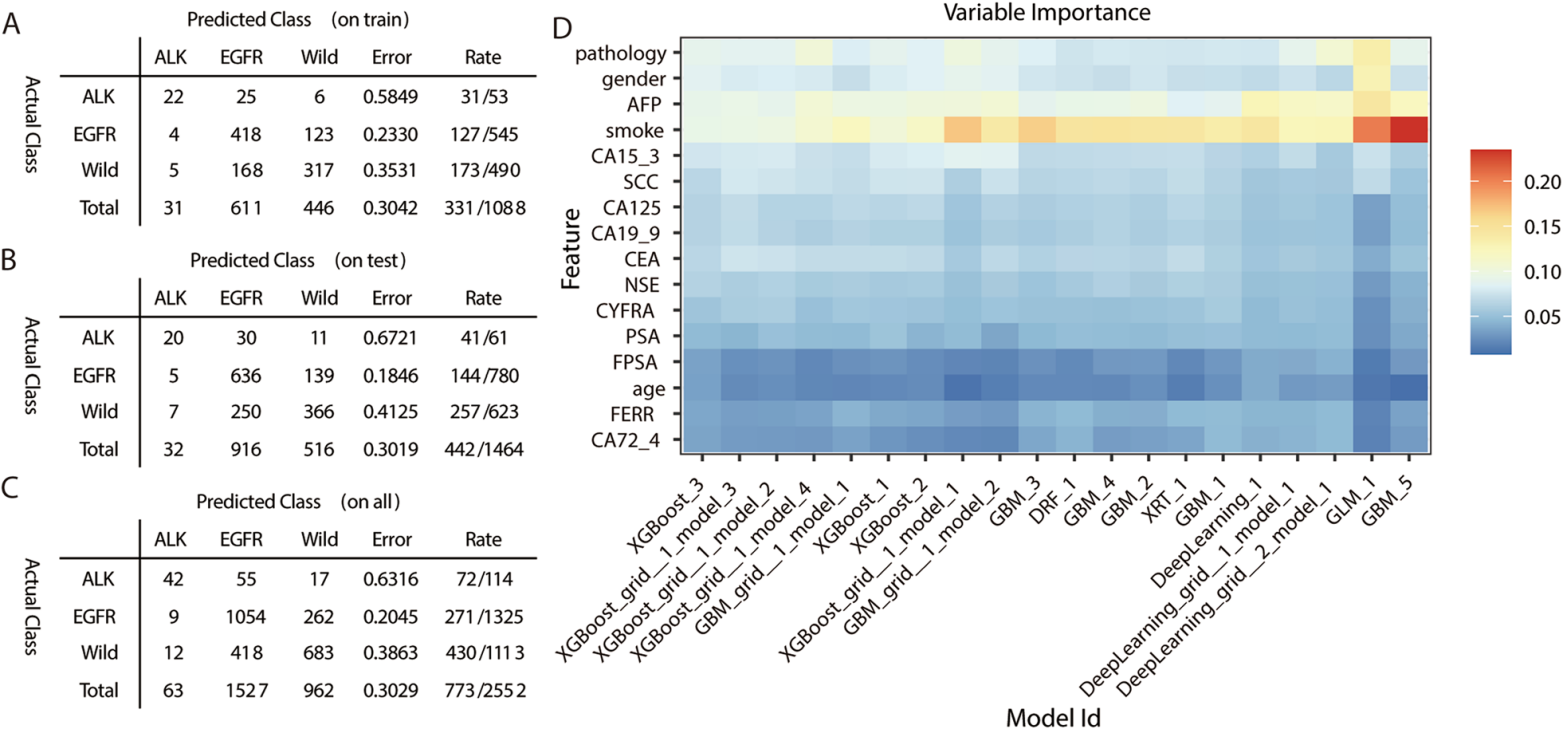
Stacked Ensemble model to distinguish ERFR mutant status and ALK rearrangement concurrently and corresponding variable importance. **A**–**C** The overall accuracy in the training cohort, the testing cohort and total patients. **D** The importance of differential clinical parameters in Stacked Ensemble model to distinguish EGFR mutant status and ALK rearrangement concurrently. The AutoML randomly generated twenty algorithms based on deep learning model and five machine learning model, and the twenty models were interpreted using feature importance plots through the matplotlib package built in the software

Although AI models yielded satisfactory efficiency in predicting EGFR or ALK status separately, to avoid statistical errors in the hypothesis test and to further facilitate clinical practice, we attempted to build a model to distinguish EGFR mutant status and ALK rearrangement concurrently. The overall accuracy in the training cohort, testing cohort, and total patients to identify ALK rearrangement and EGFR mutation status was 0.70 (Fig. 6A–C). Smoking history, pathology, and sex provided important information for this model (Fig. 6D). These results were not as satisfactory as those of the former separate models, possibly because of the mutually exclusive status between EGFR and ALK alterations, as well as the small difference between their clinical characteristics or STMs.

## Discussion

One critical trend in precision medicine for NSCLC patients is the study of predictive biomarkers [31]. The identification of EGFR mutations and ALK rearrangement status is becoming increasingly important for NSCLC patients to determine the suitability of TKI treatment. Owing to the limitations of tissue biopsy, constructing an easy-to-use model based on easily available clinical indicators has become a concern. In this study, driverless AI technology was fully applied with one DL model and five ML models developed to predict EGFR mutations and ALK positivity in NSCLC patients. These six models were stacked to develop an ensemble model that showed the best performance among all models. The stacked ensemble model yielded optimal diagnostic performance with AUCs of 0.883 and 0.921 for predicting EGFR mutation and ALK rearrangement status in the testing cohort, respectively, which demonstrated predictive accuracy in different patient populations. In addition to some demographic features, including age, sex, and smoking status, several STMs, such as CEA, CA125, CA199, NSE, SCC, and CYFRA, were also found to be associated with EGFR and ALK mutation status in NSCLC patients. The present results suggest that it is feasible to use DL and ML based on clinical features to predict EGFR and ALK mutation status in NSCLC patients.

For NSCLC patients, EGFR mutations were reported to be more frequent in never-smokers, adenocarcinomas, patients of East Asian ethnicity, and females [37]. Approximately 40% of never-smokers present with EGFR mutations [38]. Similarly, ALK-positive NSCLC patients are usually younger, female, and non-smokers [39, 40]. Phyu et al. constructed a predictive model presented as a nomogram comprising three predictors (sex, ethnicity, and smoking status) to evaluate EGFR mutation probabilities in non-squamous NSCLC patients, with a sensitivity of 68% and a specificity of 78% when the probability cut-off point was 0.2 [41]. The examination of EGFR mutations in elderly NSCLC patients is crucial, since EGFR-TKI treatments, including gefitinib and osimertinib, have been reported to be safe and effective for these patients [42, 43]. Age is an essential factor to consider when adopting EGFR-TKI treatment for EGFR mutation-positive NSCLC [44]. Our study showed that age was the most informative parameter in the DRF and XRT models, while smoking status was the most informative parameter in the GBM and XGBoost models. Age was the most important variable in both EGFR and ALK prediction models. Elevated serum CEA levels predicted the presence of EGFR mutations not only in primary lung adenocarcinoma patients, but also in patients with recurrent lung adenocarcinomas [45, 46]. Preoperative serum CEA levels are also associated with ALK fusion in patients with completely resected lung adenocarcinomas [47]. Moreover, the efficacy of EGFR-TKI treatment has been reported to be closely associated with serum CEA levels [48]. The levels of several other tumor markers, including serum CA19-9, CA24-2, and cytologic CYFRA were significantly associated with EGFR mutations in NSCLC or lung adenocarcinoma [49, 50]. Fiala et al. found that a high serum NSE level before treatment was an independent predictor of poor outcomes in NSCLC patients treated with EGFR-TKIs [51].

Currently, AI, especially ML and DL, plays an important role mainly in the field of medical image analysis and has already been applied to other medical areas due to its satisfactory application performance [52]. In digital pathology, AI methods have undertaken many image processing and classification tasks to assist in predicting disease diagnosis and the prognosis of treatment response [53]. Coudray et al. trained a DL model based on histopathological images to classify LUAD, LUSC, or normal lung tissue, and predicted EGFR mutations with an AUC of 0.754 [12]. Wang et al. developed an end-to-end DL model to predict EGFR mutation status using CT images and found a sensitivity of 72.27% and a specificity of 75.41% in validation cohorts, which were significantly higher than those in the other three models (clinical model, semantic model, and radiomics model) [13]. However, compared with complex processing procedures performed on various medical image features, demographic characteristics and levels of STMs are more easily obtained and clarified with AI approaches, despite the few studies combining them. Since the fee for this analysis of STMs is afforded by health insurance in China, its clinical applicability is considerable. Sinha et al. used demographics, vital signs, as well as laboratory and respiratory variables to develop an acute respiratory distress syndrome (ARDS) phenotypes classifier model based on the GBM algorithm, with an AUC of 0.95 in the validation cohort [54]. In the epoch of big data, ML and DL show great advantages in finding predictive models in intricate biological systems compared to conventional logistical regression. By training with a large amount of data and sifting through massive information, their reliability and efficiency are significantly improved, which is essential for medical work [55]. The AUCs of stacked ensemble models to predict EGFR mutation status and ALK rearrangement in the training and test cohorts were encouraging; however, using the same model to predict EGFR and ALK mutation status was not feasible, which is likely due to the mutually exclusive status between EGFR and ALK alterations, as well as the small difference in clinical characteristics or STMs between them [5, 40].

In addition to the easily available variables enrolled in the models, another strength of our study is that we predicted common and uncommon EGFR mutations by constructing predictive models based on several computational algorithms. Some uncommon EGFR mutations, such as T790M mutations, exon 20 insertions and so on, have been implicated in the development of resistance to TKI treatment in NSCLC patients [56, 57]. The T790M mutation can be found in < 5% of patients with NSCLC whose primary tumors were not previously treated with TKIs [58]. The PFS of patients with uncommon EGFR mutations is significantly shorter than that of patients with common EGFR mutations [59]. With the identification of uncommon EGFR mutations, higher generations of EGFR inhibitors can be chosen to overcome drug resistance to avoid ineffective therapy [60, 61].

However, the present study has certain limitations. First, our data source was derived from only one city in China, which requires validation in other populations to extend its generalizability. Second, statistical modeling based on AI technology requires a certain number of samples to obtain optimized classifiers for prediction, especially for DL, but the sample size in our study was not large enough. Third, we did not predict specific types of uncommon EGFR mutations separately, which were far more than two, due to the small proportion of each uncommon mutation of EGFR. Finally, more demographic characteristics and STMs should be included to strengthen the performance and outcomes of this study. Therefore, more large-scale prospective studies involving various populations are necessary to verify our predictive models in the future.

## Conclusion

In conclusion, this study demonstrated that ML and DL models can be useful tools to help clinicians identify EGFR mutation status and ALK rearrangement in NSCLC patients. Moreover, the identification of uncommon EGFR mutations is beneficial for evaluating the sensitivity of patients to EGFR-TKI treatment. Demographic characteristics, pathology and STMs were easily available variables used to construct predictive models in our study. The stacked ensemble model showed a more accurate predictive performance by optimally combining the DL model with the five ML models. Nevertheless, the reliability and generalizability of the computational algorithms constructed in our study require further large-scale epidemiological studies for verification.

## Supplementary Information

**Additional file 1: **Supplementary materials.

**Additional file 2: Fig. S1.** ROC curve for predicting EGFR or ALK mutations in the testing cohort. (A) ADC, never-smoker status, and negative CA 125 and SCC were predictors of EGFR mutations (B) Younger age and never-smoker status were predictors of ALK rearrangement.

**Additional file 3: Fig. S2** ROC curve of the stacked ensemble model for predicting EGFR mutations in adenocarcinoma-only group in the training and testing cohort, respectively. (A) adenocarcinoma patients in the training cohort; (B) adenocarcinoma patients in the testing cohort.

**Additional file 4: Fig. S3** The impacts of the first 5 variables on the diagnostic accuracy of the predictive models. (A) Deep leaning model; (B) DRF model; (C) GBM model; (D) GLM model; (E) XGBoost model; (F) XRF model.

## Data Availability

The data underlying this article will be shared on reasonable request to the corresponding author.

## Abbreviations

ADC: Adenocarcinoma
AFP: Alpha fetoprotein
AI: Artificial intelligence
ALK: Anaplastic lymphoma kinase
ARDS: Acute respiratory distress syndrome
AUC: Area under the curve
CA: Carbohydrate antigen
CK-7: Cytokeratin-7
CEA: Carcinoembryonic antigen
CT: Computed tomography
CTCs: Circulating tumor cells
CYFRA 21-1: Cytokeratin 19 fragments
Del19: Exon 19 deletion
DL: Deep learning
DRF: Distributed random forest
EGFR: Epidermal growth factor receptor
FERR: Ferritin
FFPE: Formalin-fixed paraffin-embedded
GBM: Gradient boosting machine
GLM: Generalized linear models
ICIs: Immune checkpoint inhibitors
IHC: Immunohistochemistry
ML: Machine learning
NGS: Next-generation sequencing
NSCLC: Non-small cell lung cancer
NSE: Neuron-specific enolase
PFS: Progression-free survival
ROC: Receiver operator characteristic
SCC: Squamous cell carcinoma antigen
PSA: Prostate specific antigen
FPSA: Free prostate specific antigen
STM: Serum tumor marker
SVM: Support vector machine
TKIs: Tyrosine kinase inhibitors
TTF-1: Thyroid transcription factor-1
XGBoost: Extreme gradient boosting
XRT: Extremely randomized trees
