# Supplementary material for: Predicting EGFR mutation, ALK rearrangement, and uncommon EGFR mutation in NSCLC patients by driverless artificial intelligence: a cohort study
Source: Respir Res. 2022 May 27;23:132. doi: 10.1186/s12931-022-02053-2 (PMC9145462; doi:10.1186/s12931-022-02053-2)

**Supplementary Methods**

**Machine learning and deep learning algorithms**

The detailed methods of each machine learning or deep learning algorithms could be available in H2O documentation site (https://docs.h2o.ai/h2o/latest-stable/h2o-docs/index.html). Essential descriptions were provided as below for your reference.

**Generalized Linear Models (GLM)**: Provides flexible generalization of ordinary linear regression for response variables with error distribution models other than a Gaussian (normal) distribution. GLM unifies various other statistical models, including Poisson, linear, logistic, and others when using l1 and l2 regularization.

Following the definitive text by P. McCullagh and J.A. Nelder (1989) on the generalization of linear models to non-linear distributions of the response variable Y, H2O fits GLM models based on the maximum likelihood estimation via iteratively reweighed least squares.


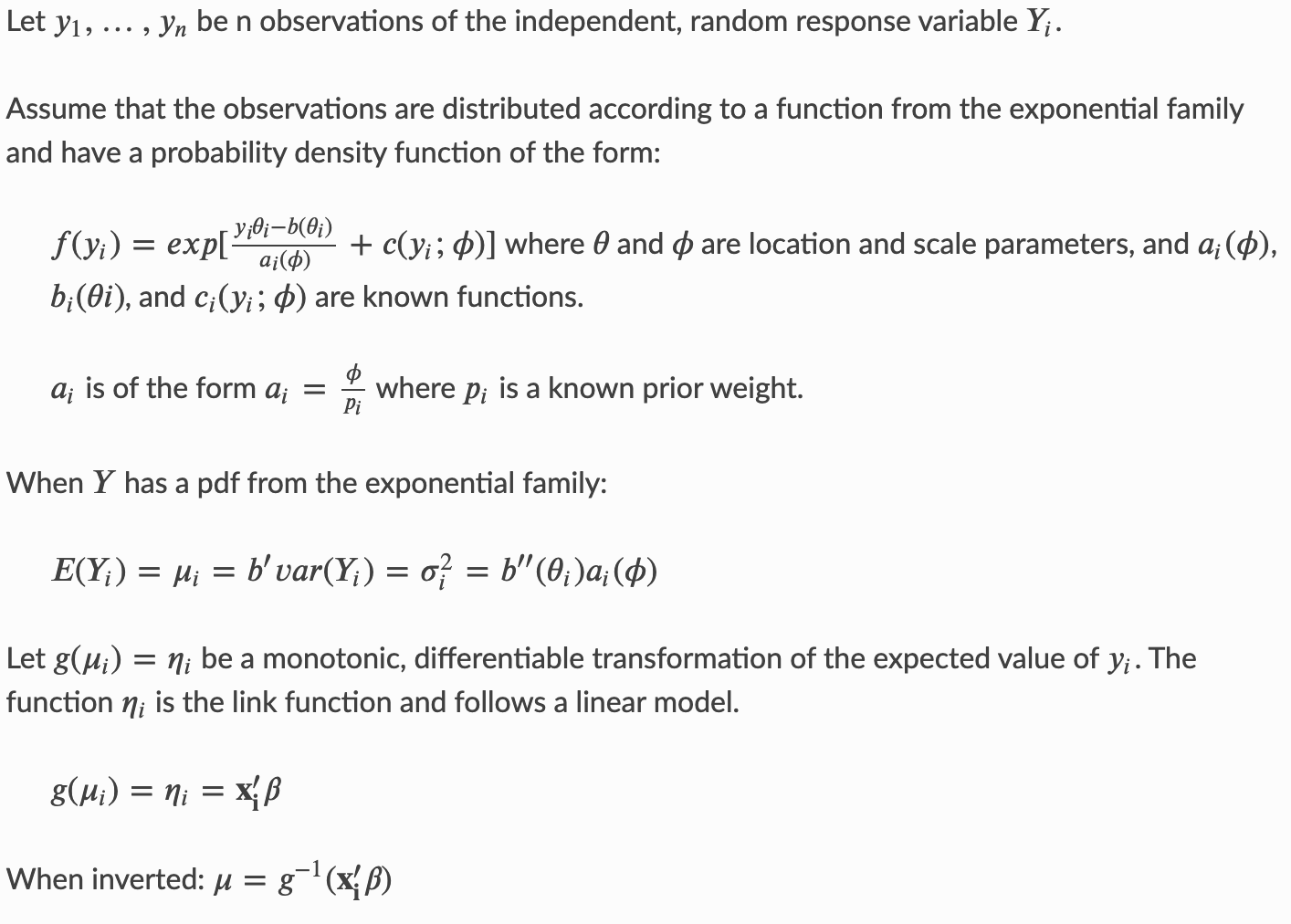


Maximum Likelihood Estimation


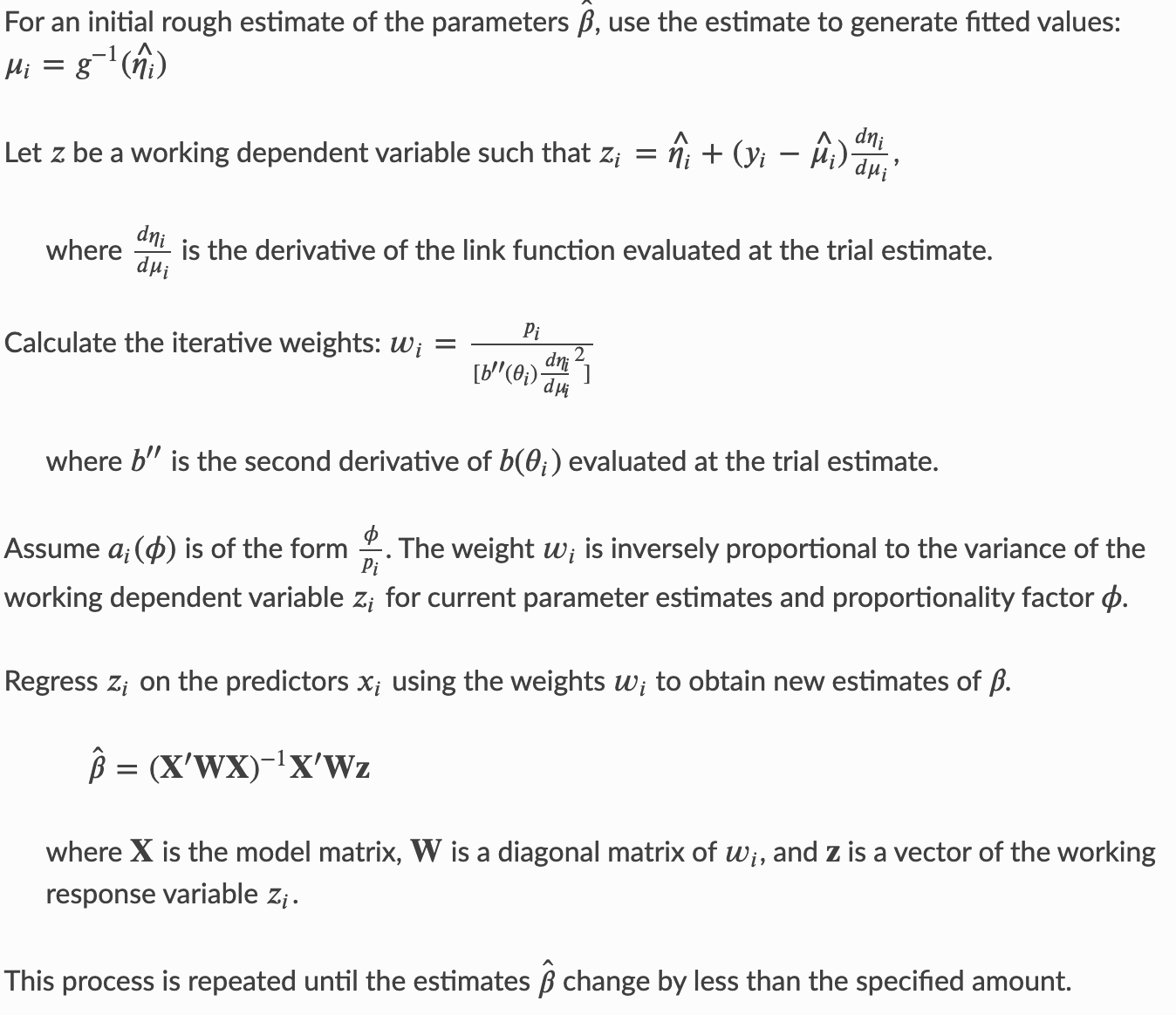


**Distributed Random Forest (DRF)**: Averages multiple decision trees, each created on different random samples of rows and columns. It is easy to use, non-linear, and provides feedback on the importance of each predictor in the model, making it one of the most robust algorithms for noisy data.

**Extremely randomized trees (XRT)**: XRT randomness goes one step further in the way that splits are computed. As in random forests, a random subset of candidate features is used, but instead of looking for the most discriminative thresholds, thresholds are drawn at random for each candidate feature, and the best of these randomly generated thresholds is picked as the splitting rule. This usually allows to reduce the variance of the model a bit more, at the expense of a slightly greater increase in bias.


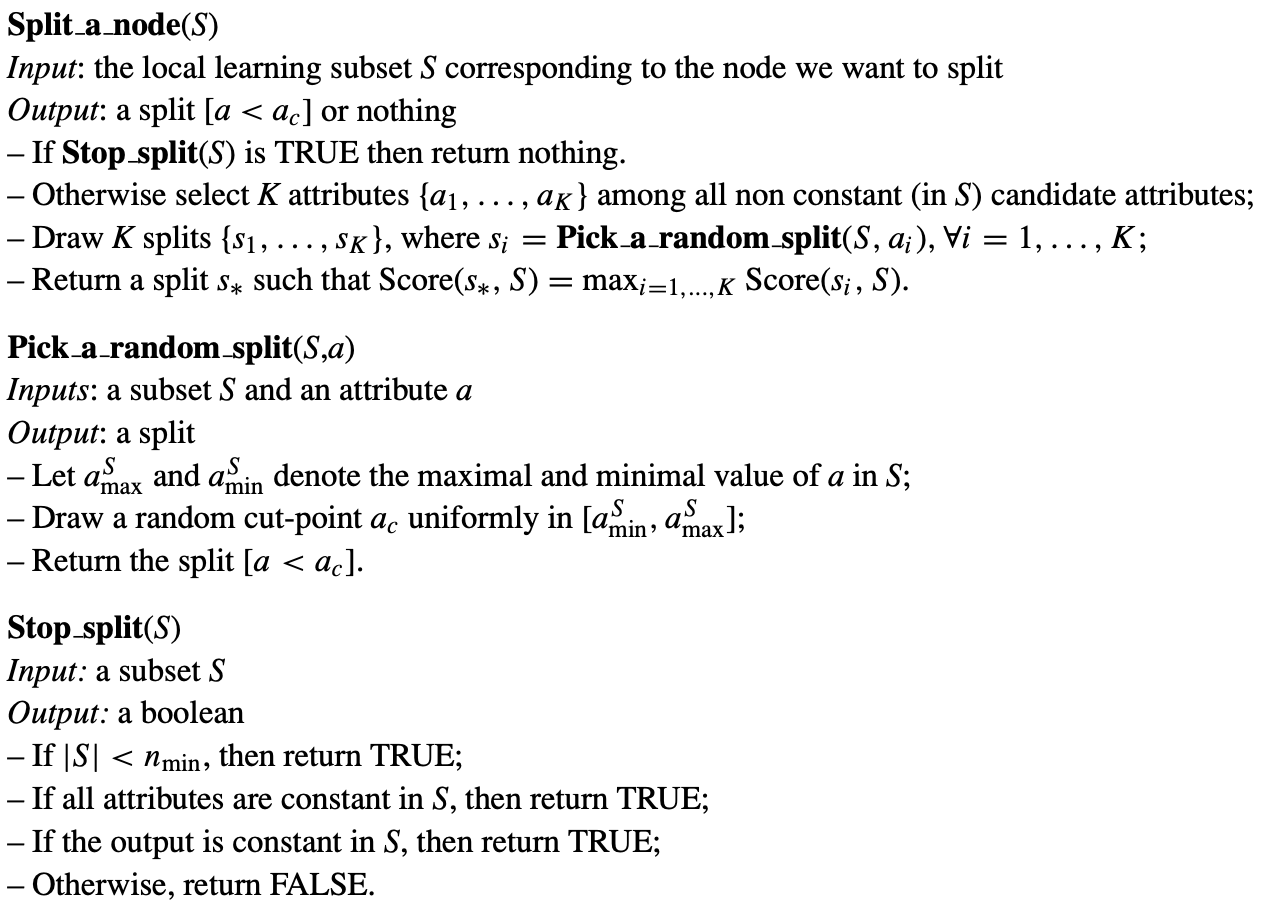


**Gradient Boosting (GBM):** Produces a prediction model in the form of an ensemble of weak prediction models. It builds the model in a stage-wise fashion and is generalized by allowing an arbitrary differentiable loss function. It is one of the most powerful methods available today.

H2O’s Gradient Boosting Algorithms follow the algorithm specified by Hastie et al (2001):


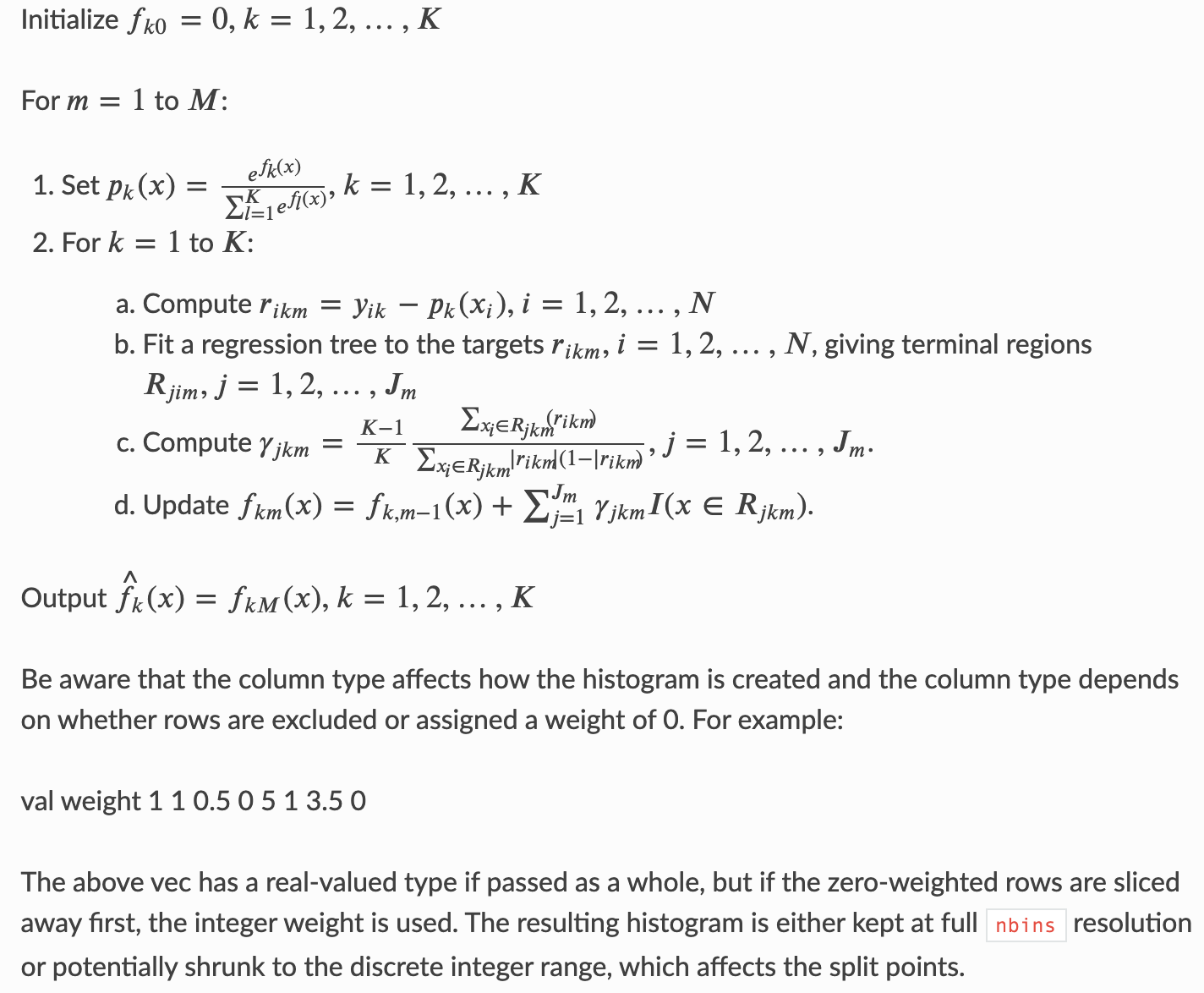


**XGBoost**: XGBoost is an optimized gradient boosting library that implements machine learning algorithms under the Gradient Boosting Machine (GBM) framework. For many problems, XGBoost is the one of the best GBM frameworks today. In other cases, the H2O GBM algorithm comes out on top. Both implementations are available on the H2O platform.

**Deep Learning**: Models high-level abstractions in data by using non-linear transformations in a layer-by-layer method. Deep learning is an example of supervised learning, which can use unlabeled data that other algorithms cannot.

H2O’s Deep Learning is based on a multi-layer feedforward artificial neural network that is trained with stochastic gradient descent using back-propagation. The network can contain a large number of hidden layers consisting of neurons with tanh, rectifier, and maxout activation functions. Advanced features such as adaptive learning rate, rate annealing, momentum training, dropout, L1 or L2 regularization, checkpointing, and grid search enable high predictive accuracy. Each compute node trains a copy of the global model parameters on its local data with multi-threading (asynchronously) and contributes periodically to the global model via model averaging across the network.


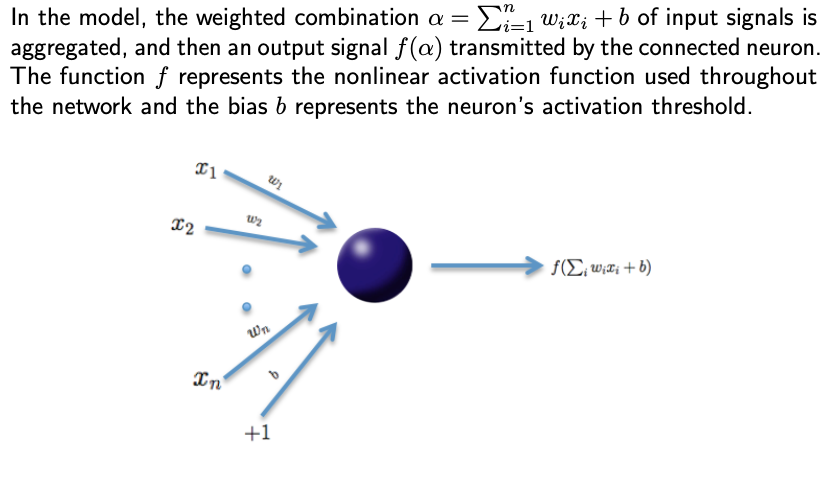


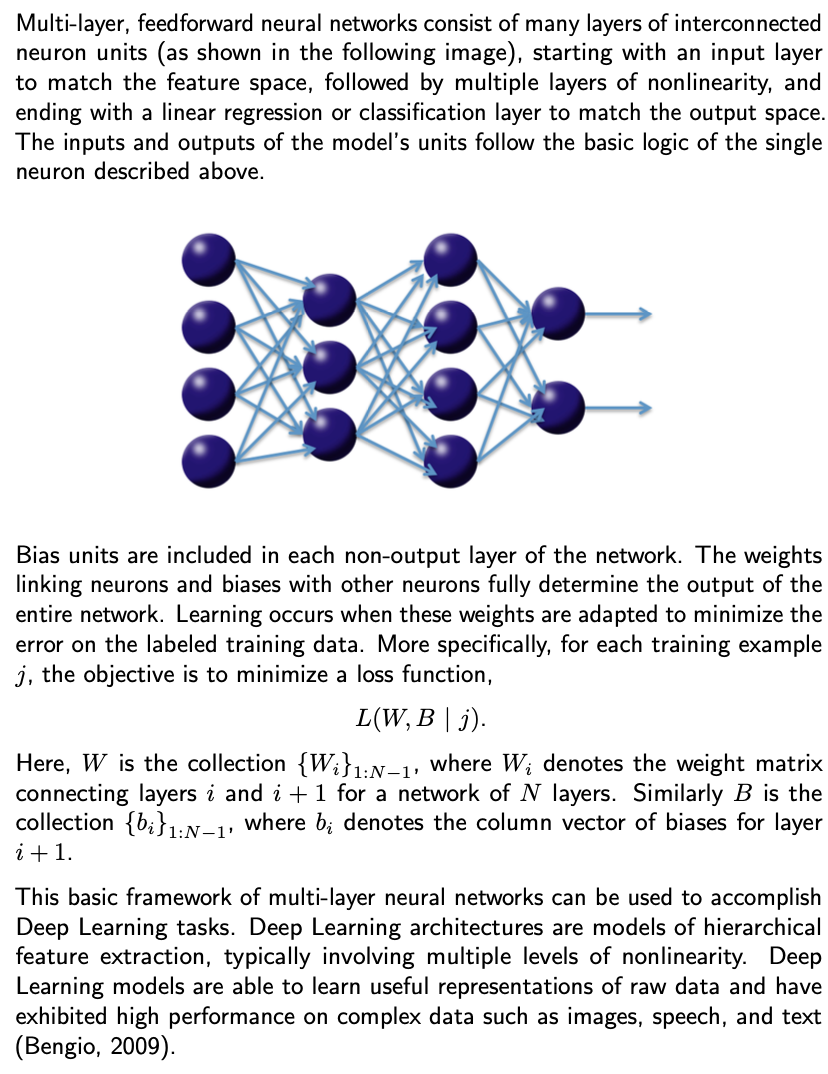

Supplement: Supplementary file 1 — Additional file 1: Supplementary materials. [file 12931_2022_2053_MOESM1_ESM.docx]
