# Supplementary figures and images for: Predicting EGFR mutation, ALK rearrangement, and uncommon EGFR mutation in NSCLC patients by driverless artificial intelligence: a cohort study
Source: Respir Res. 2022 May 27;23:132. doi: 10.1186/s12931-022-02053-2 (PMC9145462; doi:10.1186/s12931-022-02053-2)

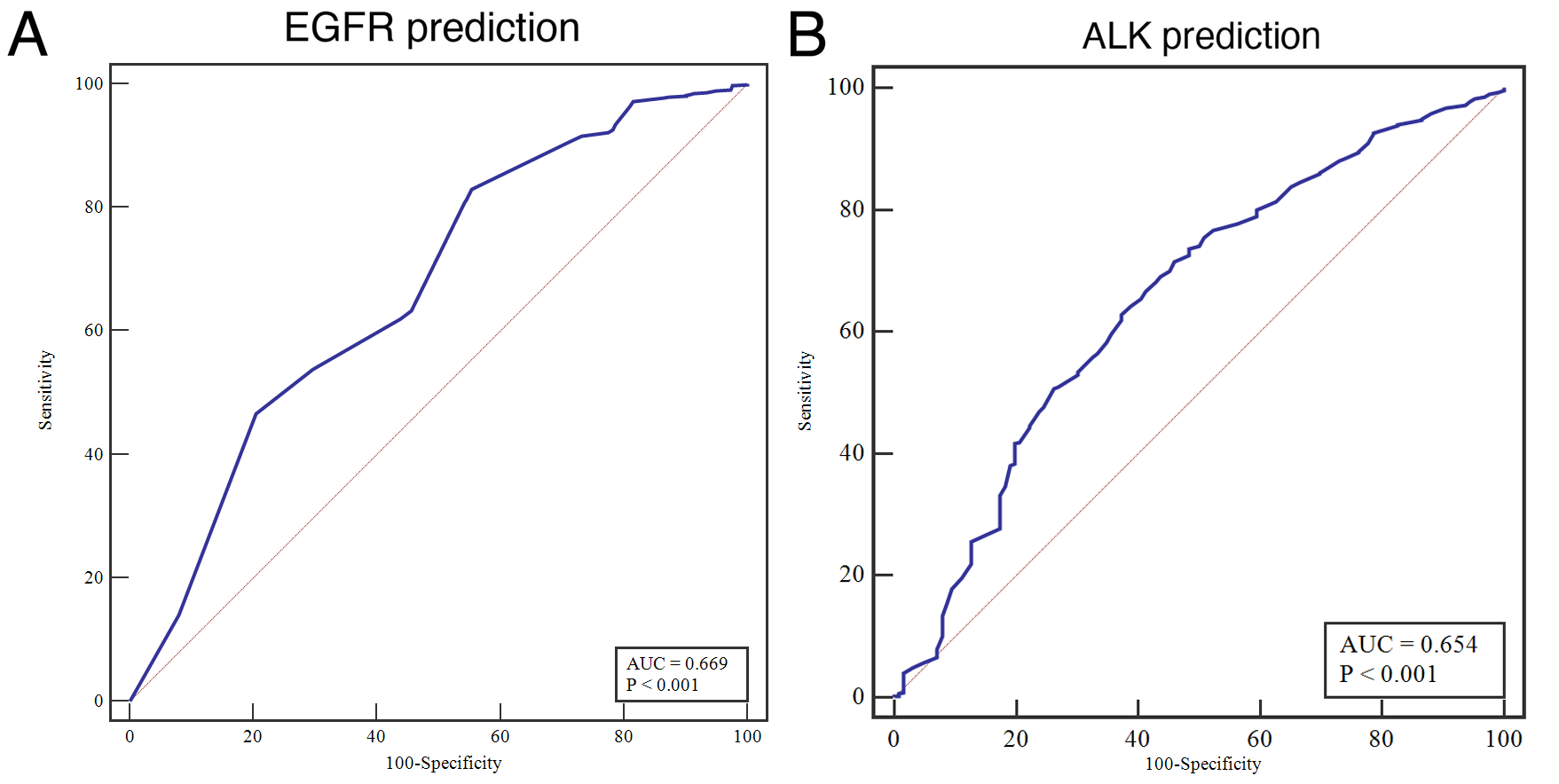

Supplement: Supplementary file 2 — Additional file 2: Fig. S1. ROC curve for predicting EGFR or ALK mutations in the testing cohort. (A) ADC, never-smoker status, and negative CA 125 and SCC were predictors of EGFR mutations (B) Younger age and never-smoker status were predictors of ALK rearrangement. [file 12931_2022_2053_MOESM2_ESM.tif]

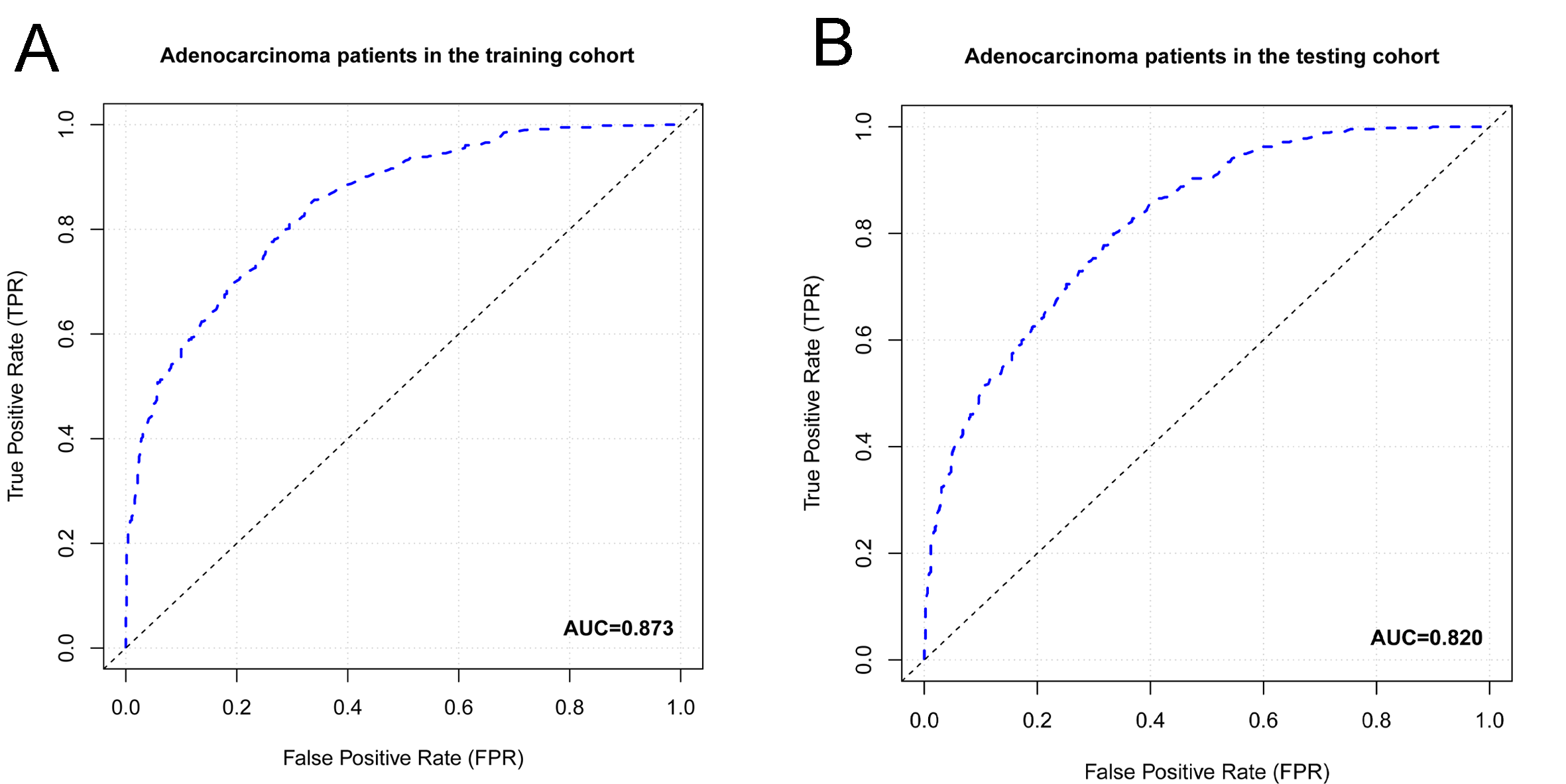

Supplement: Supplementary file 3 — Additional file 3: Fig. S2 ROC curve of the stacked ensemble model for predicting EGFR mutations in adenocarcinoma-only group in the training and testing cohort, respectively. (A) adenocarcinoma patients in the training cohort; (B) adenocarcinoma patients in the testing cohort. [file 12931_2022_2053_MOESM3_ESM.tif]

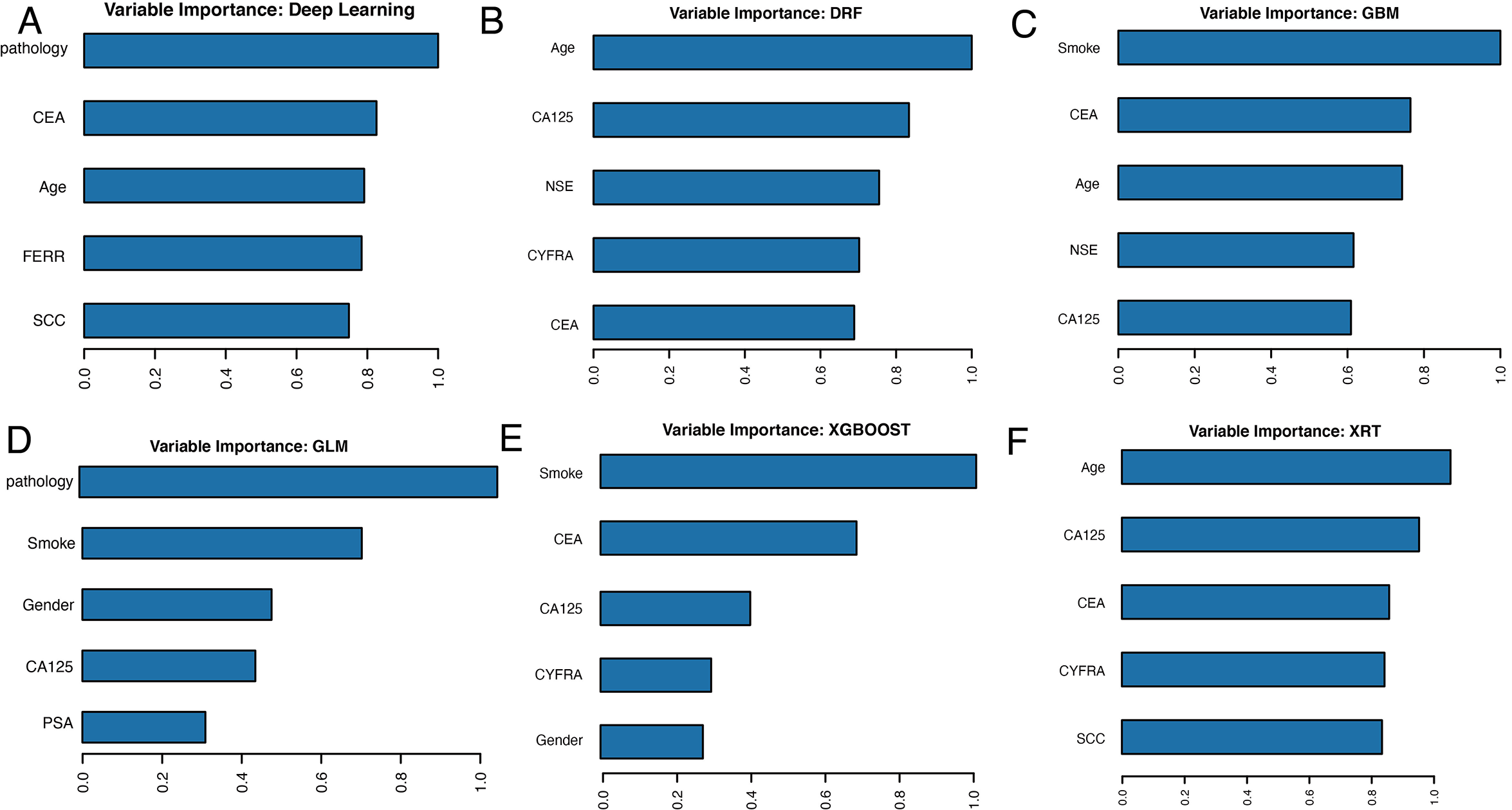

Supplement: Supplementary file 4 — Additional file 4: Fig. S3 The impacts of the first 5 variables on the diagnostic accuracy of the predictive models. (A) Deep leaning model; (B) DRF model; (C) GBM model; (D) GLM model; (E) XGBoost model; (F) XRF model. [file 12931_2022_2053_MOESM4_ESM.tif]
